# Effects of Tai Chi or Exercise on Sleep in Older Adults With Insomnia: A Randomized Clinical Trial

**DOI:** 10.1001/jamanetworkopen.2020.37199

**Published:** 2021-02-15

**Authors:** Parco M. Siu, Angus P. Yu, Bjorn T. Tam, Edwin C. Chin, Doris S. Yu, Ka-Fai Chung, Stanley S. Hui, Jean Woo, Daniel Y. Fong, Paul H. Lee, Gao X. Wei, Michael R. Irwin

**Affiliations:** 1 Division of Kinesiology, School of Public Health, Li Ka Shing Faculty of Medicine, the University of Hong Kong, Pokfulam, Hong Kong, China; 2 Department of Health, Kinesiology and Applied Physiology, Concordia University, Montreal, Canada; 3 School of Nursing, Li Ka Shing Faculty of Medicine, the University of Hong Kong, Pokfulam, Hong Kong, China; 4 Department of Psychiatry, Li Ka Shing Faculty of Medicine, the University of Hong Kong, Pokfulam, Hong Kong, China; 5 Department of Sports Science and Physical Education, Faculty of Education, the Chinese University of Hong Kong, Shatin, Hong Kong, China; 6 Department of Medicine and Therapeutics, Faculty of Medicine, the Chinese University of Hong Kong, Shatin, Hong Kong, China; 7 School of Nursing, Faculty of Health and Social Sciences, the Hong Kong Polytechnic University, Hung Hom, Hong Kong, China; 8 Institute of Psychology, Chinese Academy of Sciences, Beijing, China; 9 Cousins Center for Psychoneuroimmunology and Department of Psychiatry and Biobehavioral Sciences, University of California, Los Angeles

## Abstract

**Importance:**

Previous studies that have shown tai chi to improve sleep were mainly based on subjective assessments, which might have produced results confounded by self-reporting bias.

**Objective:**

To compare the effectiveness of tai chi for improving sleep in older adults with insomnia with conventional exercise and a passive control group using actigraphy-based objective measurements.

**Design, Setting, and Participants:**

This randomized, 3-arm, parallel group, assessor-masked clinical trial was conducted at a single research unit in Hong Kong between August 2014 and August 2018. Eligible participants, aged 60 years or older and with chronic insomnia, were randomly allocated into tai chi training, exercise, and control groups.

**Interventions:**

12-week tai chi training, 12-week conventional exercise, and no intervention control.

**Main Outcomes and Measures:**

Primary outcomes were measures taken from actigraphy sleep assessment. Secondary outcomes included remission of insomnia, insomnia treatment response, Pittsburgh Sleep Quality Index score, Insomnia Severity Index score, and self-reported sleep using a 7-day sleep diary. Assessments were performed at baseline, end of the intervention (postintervention), and 24 months after the intervention (follow-up). Data analysis was performed from September 2018 to August 2020.

**Results:**

A total of 320 participants (mean [SD] age, 67.3 [6.8] years; mean [SD] insomnia duration, 124.4 [134.5] months; 256 [80.0%] women) were randomly allocated into control (110 participants), exercise (105 participants), and tai chi (105 participants) groups and included in the data analysis. Compared with the control group, the exercise and tai chi groups showed improved sleep efficiency (exercise vs control: adjusted mean difference, +3.5%; 95% CI, 1.8-5.2; *P* < .001; tai chi vs control: adjusted mean difference, +3.4%; 95% CI, 1.6-5.1; *P* < .001) and reductions of wake time after sleep onset (exercise vs control: −17.0 minutes; 95% CI, −24.9 to −9.0; *P* < .001; tai chi vs control: −13.3 minutes; 95% CI, −21.3 to −5.2; *P* = .001) and number of awakenings (exercise vs control: −2.8 times; 95% CI, −4.0 to −1.6; *P* < .001; tai chi vs control: −2.2 times; 95% CI, −3.5 to −1.0; *P* < .001) as assessed by actigraphy at postintervention; although there were no significant differences between the exercise and tai chi groups. The actigraphy-assessed beneficial effects were maintained in both intervention groups at follow-up.

**Conclusions and Relevance:**

Conventional exercise and tai chi improved sleep and the beneficial effects sustained for 24 months, although the absolute improvements in sleep parameters were modest. Improvements in objective sleep parameters were not different between the tai chi and exercise groups, suggesting that tai chi can be an alternative approach for managing insomnia.

**Trial Registration:**

ClinicalTrials.gov Identifier: NCT02260843

## Key Points

**Question:**

Can tai chi improve sleep as effectively as conventional exercise in older adults with insomnia?

**Findings:**

In this randomized clinical trial using data collected from 320 older adults, both tai chi and conventional exercise were associated with improved sleep. Both interventions were equally effective in improving various actigraphy-assessed sleep parameters, and these beneficial effects remained persistent 24 months after the intervention with no significant differences between the 2 intervention groups.

**Meaning:**

Given that tai chi is an accepted form of physical activity among older people because of its gentle, low-impact exercises, it can represent an alternative approach to fulfill the physical activity recommendations for improving sleep for individuals who are averse to conventional exercise.

## Teaser

This randomized clinical trial assessed the effectiveness of tai chi practice for insomnia among adults aged 60 years or older in comparison with conventional exercise and a control group.

## Introduction

More than half of older adults worldwide report sleep disturbances, of whom between 20% and 40% report insomnia.^1^ Insomnia and persistent sleep disturbance impair quality of life and prospectively increase mortality and morbidity.^2,3^ The US National Health Interview Survey 2017 data reported that 29.8% of individuals with sleep problems used mind-body medicine to improve sleep.^4^

Tai chi is a mind-body exercise known to confer a variety of health benefits that includes improvement of self-reported sleep quality.^5,6,7^ While subjective data are valuable for the evaluation of insomnia treatment response, the placebo effect may compromise the reliability of data.^8,9^ Therefore, it is essential to include both subjective and objective measurements for accurate evaluation of insomnia treatment effectiveness. Considering the conclusion drawn by most studies that tai chi improves sleep is largely based on subjective measurements,^10^ objective assessments are necessary to confirm the self-reported data.

To validate the use of tai chi as an alternative approach for managing insomnia, this study aimed to (1) examine the effectiveness of tai chi on improving objective sleep in older adults with insomnia relative to a passive control using actigraphy and (2) compare the effectiveness of tai chi with conventional exercise. With both subjective and objective data, the effects of tai chi on sleep improvement can be more comprehensively analyzed, which will shed light on its future establishment as a nonpharmacological approach for insomnia management. We hypothesized that a 12-week tai chi training would induce significantly larger improvements in objective sleep parameters than the control and its conventional exercise counterpart.

## Methods

This study adhered to the following Consolidated Standards of Reporting Trials (CONSORT) reporting guidelines: Consolidated Standards of Reporting Trials-Patient Reported Outcomes (CONSORT-PRO),^11^ CONSORT extension for multi-arm trials,^12^ and CONSORT extension for nonpharmacologic trials.^13^ This study also followed the template for intervention description and replication (TIDieR) guide.^14^ A detailed description of the methods is provided in the trial protocol (Supplement 1). Written informed consent was obtained before the start of the study. All experimental procedures involving human research received ethics approval from the Hong Kong Polytechnic University human subjects ethics office.

### Participants

This was a randomized, 3-arm, parallel, assessor-masked clinical trial conducted in a single center in Hong Kong between August 2014 and August 2018. Participants were recruited through promotion in community centers, elderly day care centers, housing estates, and local universities. This study involved 320 Chinese adults aged 60 years or older with chronic insomnia diagnosed according to the *Diagnostic and Statistical Manual of Mental Disorders* (Fifth Edition) (*DSM-5*).^15^ Exclusion criteria included: (1) regular practice of moderate-intensity exercise or tai chi (ie, >3 times/week and >30 minute/session), (2) any physical disability that precluded participation in the interventions, and (3) major confounding conditions known to induce sleep perturbations, such as severe chronic diseases (eg, cancer, autoimmune disease) and related treatments (eg, cancer chemotherapy) or chronic pain disorders.

### Randomization and Masking

Randomization was performed by independent research personnel using an automated permuted block algorithm with a block size of 30. All allocation sequences were concealed from the researcher responsible for participant enrollment. Assessments were conducted at baseline, the end of intervention (postintervention), and 24 months after the intervention (follow-up). The outcome assessors were masked to the participant’s group allocation. All instructors involved in the conventional exercise and tai chi classes were not masked to group allocation and the intervention delivered because of the intervention nature. The details of the randomization and masking procedures were described in the supplementary study protocol (Supplement 1).

### Intervention

Participants in the control group received no intervention in addition to their preexisting usual care, except monthly phone calls to record their sleep conditions. Participants in the exercise group attended a 12-week conventional exercise training program, which consisted of brisk walking and muscle-strengthening exercises. Participants in the tai chi group attended a 12-week Yang-style 24-form tai chi training program, which is the tai chi form/style most commonly adopted and studied in the literature. Both interventions consisted of three 1-hour training sessions per week. The details of each intervention are described in eTable 1 in Supplement 2. Training sessions of conventional exercise and tai chi were delivered by certified instructors with at least 5 years of experience in coaching the respective intervention. The training sessions were conducted in small groups (ie, 6-15 participants with both genders in the group). Our research personnel made occasional visits to the sessions to ensure the instructions were delivered in accordance to our protocol.

### Sample Size Estimation

The effect size of tai chi intervention on sleep parameters assessed objectively or subjectively in the 7-day sleep diary was not available by the time this study was designed. As this study employed a 3-arm pretest-posttest design, estimation of sample size was performed using G^*^Power 3.0 by setting the test family and statistical test sections as “F tests” and “ANOVA: Repeated measures, within-between interaction.” By using an interaction Cohen *d* = 0.22, calculated based on previously reported improvement in subjective sleep quality in the Pittsburgh sleep quality index (PSQI),^7^ 81 participants were needed to achieve a 80% statistical power (α = .05).

### Outcome Measures

The primary outcome was sleep quality assessed by actigraphy. Participants were instructed to wear a wrist actigraph (Actigraph model wGT3X-BT) on the nondominant wrist for 24 hours over a course of 7 days. The actigraph objectively measured the 7-day average of sleep parameters, including sleep efficiency, wake time after sleep onset, number of awakenings per night, sleep onset latency, total sleep time, and average wake time per awakening using the Cole-Kripke algorithm provided by the manufacturer’s software (Actilife, version 6.11.7).

Secondary outcomes included the remission of insomnia, insomnia treatment response, perceived sleep quality, insomnia severity, self-reported sleep parameters, and the use of hypnotic medication. Insomnia remission was evaluated by a masked assessor using a semistructured interview. Remission was defined as when the participant no longer met the *DSM-5* criteria for chronic insomnia. Insomnia treatment response was defined by a decrease in PSQI by at least 5 points, which indicates moderate clinically meaningful attenuation of insomnia symptoms.^5^ Estimation of perceived sleep quality and insomnia severity were assessed by the PSQI and insomnia severity index (ISI) respectively. Participants were instructed to record their sleep patterns, including bedtime, sleep rising time, wake time after sleep onset, total sleep time, number of awakenings, and sleep onset latency daily in a 7-day sleep diary. Sleep efficiency was estimated by (total sleep time / total time in bed) × 100%. Average awaken time was estimated by (wake time after sleep onset / number of awakenings). The dosage and frequency of any hypnotic medication were also recorded in the 7-day sleep diary. We converted the medication consumed into the lowest recommended dosage (LRD) units, as defined by the Prescribers’ Digital Reference and presented the data as the weekly consumed LRD.^16^

### Statistical Analysis

Data were analyzed between September 2018 and August 2020, and expressed as mean (SD) measures. Intention-to-treat analysis was employed. Data from actigraphy measurements, the 7-day sleep diary, PSQI, and ISI were analyzed by a generalized estimated equation (GEE) model using group and time as main effects and baseline as a covariate. A pairwise comparison was performed to compare the differences between the intervention groups whenever a group × time interaction effect was observed. Holm correction was employed in the pairwise comparison among the primary outcomes (ie, the actigraphy measurements) in order to account for multiplicity. Pairwise comparisons of the secondary outcomes were performed using a closed test procedure with Holm-Bonferroni correction. Missing values were not imputed, as GEE can accommodate missing data and provides a natural way to deal with missing values.^17^ A subgroup analysis was conducted in participants taking hypnotic medication to examine changes in hypnotic medication usage. Insomnia remission rate and treatment response rate were examined by logistic regression, followed by linear contrast for pairwise comparison analysis. Statistical significance was accepted in 2-sided tests at *P* < .05.

## Results

### Baseline Characteristics of Participants

**Figure 1.**
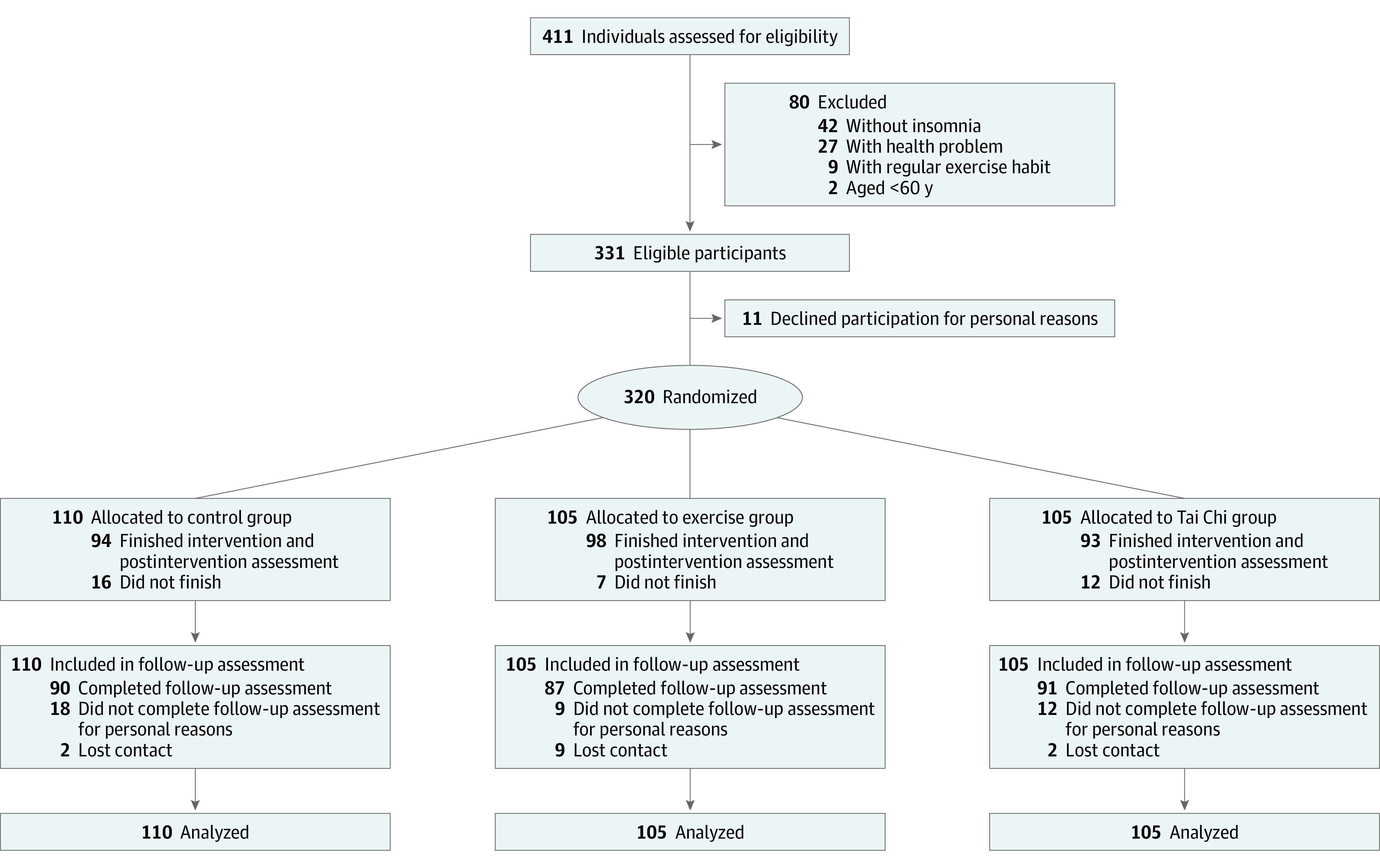
Schematic Presentation of the Participants in the Screening, Randomization, and Intervention Stages

A total of 411 individuals were screened for chronic insomnia by semistructured interviews (Figure 1) from August 2014. Overall, 320 eligible participants (256 [80.0%] women; mean [SD] age, 67.3 [6.8] years; insomnia duration, 124.4 [134.5] months) were randomly assigned to the control (110 participants), exercise (105 participants), and tai chi (105 participants) treatment groups within 1 month after the baseline assessment. Data of all participants were included in the data analysis. No participants exhibited any coexisting psychiatric disorder in addition to chronic insomnia during the experimental period. A history of major depressive disorder was found in 7 participants (control, 3 participants; exercise, 2 participants; tai chi, 2 participants) but had reached remission for at least a year before participation—none of these participants reported relapse of the disorder throughout the study. No participants exhibited a history of substance abuse or other sleeping disorders, and no participants had previously received any nonpharmacological treatments of chronic insomnia. A total of 60 participants were taking hypnotic medications. A total of 285 participants (control, 94; exercise, 98; tai chi, 93) completed the postassessment, and 268 participants (control, 90; exercise, 87; tai chi, 91) completed the follow-up assessment before August 2018. The rate of adherence, measured as the class attendance, was not significantly different between the exercise and tai chi groups (72.0% and 72.4%, respectively). No adverse events were observed. The baseline characteristics of participants are summarized in Table 1.

**Table 1.** Summary of Baseline Characteristics of Participants

Characteristic	Participants, mean (SD)		
	Control (n = 110)	Exercise (n = 105)	Tai chi (n = 105)
Age, y	68.0 (8.2)	67.3 (5.7)	66.5 (6.4)
Insomnia duration, mo	130.7 (130.9)	120.3 (141.7)	121.8 (132.0)
Female, No. (%)	88 (80.0)	84 (80.0)	84 (80.0)
Actigraphy			
Sleep efficiency, %	80.8 (10.2)	82.6 (7.5)	82.4 (8.1)
Wake time after sleep onset, min	82.1 (51.2)	79.0 (37.8)	76.6 (36.3)
No. of awakenings	17.5 (6.5)	18.8 (7.0)	19.0 (7.1)
Sleep onset latency, min	7.2 (7.1)	5.6 (8.3)	5.7 (5.6)
Total sleep time, min	372.5 (77.5)	388.5 (66.7)	396.4 (69.9)
Average awaken time, min	4.9 (3.4)	4.8 (5.3)	4.1 (1.6)
Sleep questionnaire			
PSQI	11.4 (3.2)	11.4 (2.9)	11.3 (2.9)
ISI	13.7 (5.2)	13.4 (5.1)	14.0 (4.0)
Sleep diary			
Sleep efficiency, %	66.9 (32.5)	62.7 (18.0)	64.3 (18.4)
Wake time after sleep onset, min	89.8 (88.8)	99.3 (82.2)	94.8 (76.5)
No. of awakenings	1.9 (1.3)	2.1 (1.4)	2.1 (1.2)
Sleep onset latency, min	66.9 (65.7)	66.8 (52.2)	63.5 (48.8)
Total sleep time, min	266.6 (94.3)	262.7 (78.1)	275.0 (98.2)
Average awaken time, min	57.5 (64.8)	63.6 (76.1)	55.2 (62.4)
Hypnotic medication usage, LRD units/wk	8.5 (7.7)	8.1 (5.2)	7.7 (6.6)
History of psychiatric diseases			
Major depressive disorder	3	2	2
Anxiety disorder	0	0	0
Substance abuse	0	0	0
Other sleep disorders	0	0	0
Coexisting psychiatric diseases			
Major depressive disorder	0	0	0
Anxiety disorder	0	0	0
Substance abuse	0	0	0
Other sleep disorders	0	0	0

Abbreviations: ISI, insomnia severity index; LRD, lowest recommended dosage; PSQI, Pittsburgh sleep quality index.

### Primary Outcomes

Interaction effects were observed in actigraphy-assessed sleep efficiency, wake time after sleep onset, number of awakenings, and sleep onset latency. Compared with the control group, both the exercise and tai chi groups showed an increase in sleep efficiency at postintervention (exercise vs control: adjusted mean difference, +3.5%; 95% CI, 1.8-5.2; *P* < .001; tai chi vs control: adjusted mean difference, +3.4%; 95% CI, 1.6-5.1; *P* < .001) and follow-up (exercise vs control: adjusted mean difference, +3.3%; 95% CI, 1.4-5.2; *P* < .001; tai chi vs control: adjusted mean difference, +6.2%; 95% CI, 4.4-8.1; *P* < .001). Although there were no significant differences between the exercise and tai chi groups at postintervention, the increase in the tai chi group was more noticeable than the exercise group at the follow-up (tai chi vs exercise: adjusted mean difference, +2.9%; 95% CI, 1.1-4.8; *P* = .002) (Table 2 and Table 3). Compared with the control group, both the exercise and tai chi groups showed a reduction of wake time after sleep onset at the postintervention (exercise vs control: −17.0 minutes; 95% CI, −24.9 to −9.0; *P* < .001; tai chi vs control: −13.3 minutes; 95% CI, −21.3 to −5.2; *P* = .001) and follow-up (exercise vs control: −23.0 minutes; 95% CI, −31.7 to −14.2; *P* < .001; tai chi vs control: −27.1 minutes; 95% CI, −35.8 to −18.4; *P* < .001), with no significant differences between the exercise and tai chi groups at both assessments (Table 2 and Table 3). Compared with the control group, the number of awakenings at postintervention was reduced in both the exercise and tai chi groups (exercise vs control: −2.8 times; 95% CI, −4.0 to −1.6; *P* < .001; tai chi vs control: −2.2 times; 95% CI, −3.5 to −1.0; *P* < .001), but there were no significant differences between the intervention groups (Table 2 and Table 3). Although both interventions led to a reduced number of awakenings compared with the control group at follow-up, only the reduction in the tai chi group reached statistical significance (tai chi vs control: −2.8 times; 95% CI, −4.1 to −1.4; *P* < .001). However, the improvement associated with tai chi was not robust enough to yield a significant difference relative to conventional exercise (Table 2 and Table 3). There was no significant difference in sleep onset latency among the 3 groups at postintervention. At follow-up, only tai chi induced significant reduction in sleep onset latency compared with the control group (tai chi vs control: −3.8 times; 95% CI, −5.8 to −1.9; *P* < .001). By contrast, the change in the tai chi group was not robust enough to yield a significant difference relative to exercise (Table 2 and Table 3). There were no significant interaction effects in actigraphy-assessed total sleep time or average awaken time. (Table 2 and Table 3).

**Table 2.** Summary of Generalized Estimated Equation Analysis

Measurement	Mean (SD)			*P* value^a^		
	Baseline	Postintervention	Follow-up	Interaction effect	Group effect	Time effect
**Primary outcomes (actigraphy-assessed)**						
Sleep efficiency, %						
Control	80.8 (10.2)	79.5 (9.6)	77.2 (9.5)	<.001	.03	<.001
Exercise	82.6 (7.5)	84.5 (5.7)	82.5 (8.8)			
Tai chi	82.4 (8.1)	84.2 (6.2)	84.9 (7.1)			
Wake time after sleep onset, min						
Control	82.1 (51.2)	89.7 (48.4)	100.1 (55.3)	<.001	.02	.001
Exercise	79.0 (37.8)	68.4 (30.5)	70.3 (28.9)			
Tai chi	76.6 (36.3)	70.8 (37.0)	65.5 (33.5)			
No. of awakenings						
Control	17.5 (6.5)	17.8 (6.8)	18.9 (6.7)	<.001	.01	.01
Exercise	18.8 (7.0)	15.7 (7.1)	17.3 (6.5)			
Tai chi	19.0 (7.1)	16.4 (6.3)	16.6 (6.8)			
Sleep onset latency, min						
Control	7.2 (7.1)	8.8 (8.0)	9.9 (9.0)	.006	.28	.003
Exercise	5.6 (8.3)	7.3 (7.8)	6.3 (8.5)			
Tai chi	5.7 (5.5)	6.2 (5.3)	5.0 (4.9)			
Total sleep time, min						
Control	372.5 (77.5)	380.5 (78.7)	357.3 (74.8)	.71	.58	.30
Exercise	388.5 (66.7)	401.3 (62.2)	387.4 (62.9)			
Tai chi	396.4 (69.9)	404.5 (69.5)	382.4 (67.3)			
Average awaken time, min						
Control	4.9 (3.4)	5.5 (3.0)	5.4 (2.6)	.44	.20	.62
Exercise	4.8 (5.3)	5.1 (3.2)	4.4 (2.2)			
Tai chi	4.1 (1.6)	4.8 (3.3)	5.2 (7.8)			
**Secondary outcomes**						
PSQI						
Control	11.4 (3.2)	10.9 (3.5)	10.0 (3.8)	.004	.72	.21
Exercise	11.4 (2.9)	9.7 (3.8)	8.9 (4.0)			
Tai chi	11.3 (2.9)	7.9 (3.8)	8.2 (3.8)			
ISI						
Control	13.7 (5.2)	12.9 (5.6)	11.3 (5.2)	<.001	.24	.11
Exercise	13.4 (5.1)	10.6 (5.8)	9.1 (5.3)			
Tai chi	14.0 (4.0)	9.1 (5.1)	8.9 (5.6)			
Sleep efficiency in 7-d sleep diary, %^b^						
Control	66.9 (32.5)	66.9 (20.4)	72.0 (18.1)	<.001	.049	.95
Exercise	62.7 (18.0)	67.1 (17.6)	78.2 (13.6)			
Tai chi	64.3 (18.4)	74.0 (19.2)	80.4 (16.6)			
Wake time after sleep onset in 7-d sleep diary, min^b^						
Control	89.8 (88.8)	84.2 (84.0)	66.2 (69.3)	.02	.50	.24
Exercise	99.3 (82.2)	80.3 (78.6)	49.4 (51.9)			
Tai chi	94.8 (76.5)	48.0 (56.3)	46.3 (59.1)			
Total sleep time in 7-d sleep diary, min^b^						
Control	269.6 (94.3)	284.0 (97.2)	293.0 (91.3)	.01	.49	.46
Exercise	262.7 (78.1)	290.7 (88.7)	326.4 (80.9)			
Tai chi	275.0 (98.2)	336.1 (101.2)	332.3 (91.2)			
No. of awakenings in 7-d sleep diary^b^						
Control	1.9 (1.3)	1.7 (1.2)	1.6 (1.1)	.07	.18	.61
Exercise	2.1 (1.4)	2.0 (1.3)	1.7 (1.1)			
Tai chi	2.1 (1.2)	1.6 (1.2)	1.5 (1.2)			
Sleep onset latency in 7-d sleep diary, min^b^						
Control	66.9 (65.7)	70.9 (71.3)	50.2 (49.3)	.12	.98	.21
Exercise	66.8 (52.2)	62.4 (51.2)	44.2 (31.3)			
Tai chi	63.5 (48.8)	46.1 (49.2)	38.0 (41.5)			
Average awaken time in 7-d sleep diary, min^b^						
Control	57.5 (64.8)	60.3 (76.7)	43.1 (51.9)	.22	.68	.10
Exercise	63.6 (76.1)	51.0 (70.2)	31.1 (34.3)			
Tai chi	55.2 (62.4)	28.4 (41.1)	29.2 (40.6)			
Hypnotic medication usage recorded in 7-d sleep diary, LRD units/wk						
Control (n = 24)	8.5 (7.7)	8.5 (7.6)	8.6 (7.8)	<.001	.05	.04
Exercise (n = 17)	8.1 (5.2)	6.5 (6.4)	5.9 (7.2)			
Tai chi (n = 19)	7.7 (6.6)	2.9 (6.1)	3.1 (5.9)			

Abbreviations: ISI, insomnia severity index; LRD, lowest recommended dosage; PSQI, Pittsburgh sleep quality index.

^a^ Generalized estimated equation model, with baseline measurement as the covariate, was used to analyze the data.

^b^ Mean values across 7-day sleep diary.

**Table 3.** Summary of Post Hoc Analysis of Generalized Estimated Equation Analysis^a^

Measurement	Postintervention			Follow-up		
	Mean adjusted change (95% CI)	*P* value	Effect size	Mean adjusted change (95% CI)	*P* value	Effect size
**Primary outcomes (actigraphy-assessed)**^b^						
Sleep efficiency, %						
Exercise vs control	3.5 (1.8 to 5.2)	<.001^c^	0.42	3.3 (1.4 to 5.2)	<.001^c^	0.35
Tai chi vs control	3.4 (1.6 to 5.1)	<.001^c^	0.39	6.2 (4.4 to 8.1)^d^	<.001^c^	0.69
Tai chi vs exercise	0.2 (−1.5 to 1.9)	.85	0.03	2.9 (1.1 to 4.8)	.002^b^	0.35
Wake time after sleep onset, min						
Exercise vs control	−17.0 (−24.9 to −9.0)	<.001^c^	0.46	−23.0 (−31.7 to −14.2)^d^	<.001^c^	0.59
Tai chi vs control	−13.3 (−21.3 to −5.2)	.001^c^	0.31	−27.1 (−35.8 to −18.4)^d^	<.001^c^	0.65
Tai chi vs exercise	3.7 (−4.2 to 11.7)	.36	0.15	−4.1 (−12.6 to 4.4)	.34	0.06
No. of awakenings						
Exercise vs control	−2.8 (−4.0 to −1.6)^d^	<.001^c^	0.48	−2.0 (−3.3 to −0.6)^d^	.004	0.44
Tai chi vs control	−2.2 (−3.5 to −1.0)	<.001^c^	0.42	−2.8 (−4.1 to −1.4)^d^	<.001^c^	0.55
Tai chi vs exercise	0.6 (−0.6 to 1.8)^d^	.35	0.05	−0.8 (−2.1 to 0.5)	.24	0.11
Sleep onset latency, min						
Exercise vs control	−0.4 (−2.2 to 1.4)	.68	0.0002	−2.5 (−4.5 to −0.6)	.01	0.24
Tai chi vs control	−1.4 (−3.2 to 0.4)	.12	0.12	−3.8 (−5.8 to −1.9)	<.001^c^	0.47
Tai chi vs exercise	−1.1 (−2.8 to 0.7)	.24	0.12	−1.3 (−3.2 to 0.6)	.17	0.23
**Secondary outcomes**^e^						
PSQI						
Exercise vs control	−1.1 (−1.9 to −0.3)	.01	0.31	−0.9 (−1.8 to −0.1)	.03	0.29
Tai chi vs control	−2.9 (−3.6 to −2.1)	<.001	0.83	−1.6 (−2.4 to −0.8)	<.001	0.49
Tai chi vs exercise	−1.8 (−2.5 to −1.0)	<.001	0.52	−0.6 (−1.4 to 0.2)	.17	0.2
ISI						
Exercise vs control	−2.2 (−3.3 to −1.0)	<.001	0.35	−2.1 (−3.2 to −0.9)	<.001	0.37
Tai chi vs control	−4.1 (−5.2 to −3.0)	<.001	0.92	−2.6 (−3.8 to −1.5)	<.001	0.61
Tai chi vs exercise	−1.9 (−3.0 to −0.8)	.001	0.57	−0.5 (−1.7 to 0.6)	.43	0.24
Sleep efficiency in 7-d sleep diary, %						
Exercise vs control	3.4 (−0.8 to 7.6)	.16	0.25	9.5 (5.1 to 13.9)	<.001	0.77
Tai chi vs control	9.0 (4.7 to 13.3)	<.001	0.51	10.2 (5.8 to 14.5)^d^	<.001	0.72
Tai chi vs exercise	5.6 (1.4 to 9.8)	.02	0.27	0.7 (−3.7 to 5.1)	.77	0.05
Wake time after sleep onset in 7-d sleep diary, min						
Exercise vs control	−12.2 (−26.8 to 2.4)	.14	0.17	−23.9 (−39.1 to −8.6)	.004	0.43
Tai chi vs control	−40.4 (−55.2 to −25.7)^d^	<.001	0.63	−22.5 (−37.5 to −7.5)	.006	0.41
Tai chi vs exercise	−28.2 (−42.8 to −13.6)	<.001	0.46	1.3 (−13.8 to 16.5)	.87	0.02
Total sleep time in 7-d sleep diary, min						
Exercise vs control	15.8 (−2.5 to 34.1)	.13	0.18	44.2 (25.2 to 63.3)^d^	<.001	0.55
Tai chi vs control	49.8 (31.3 to 68.3)^d^	<.001	0.46	36.1 (17.2 to 54.9)^d^	<.001	0.35
Tai chi vs exercise	34.0 (15.6 to 52.3)^d^	<.001	0.28	−8.1 (−27.2 to 10.9)	.48	0.2
Hypnotic medication usage recorded in 7-d sleep diary (weekly consumed LRD units)						
Exercise vs control	−2.1 (−0.6 to −3.7)	.02	0.28	−3.3 (−1.7 to −4.9)	<.001	0.36
Tai chi vs control	−4.0 (−2.5 to −5.5)	<.001	0.76	−3.7 (−2.3 to −5.2)	<.001	0.73
Tai chi vs exercise	−1.9 (−0.2 to −3.6)	.05	0.48	−0.5 (−2.2 to 1.2)	.86	0.38

Abbreviations: ISI, insomnia severity index; LRD, lowest recommended dosage; PSQI, Pittsburgh sleep quality index.

^a^ Holm correction was used for post hoc analysis in order to account for the multiplicity within an actigraphy-assessed sleep parameter among difference groups and among the actigraphy-assessed sleep parameters. Closed test procedure with Holm-Bonferroni correction was used for the post hoc analysis of the secondary outcomes.

^b^ No effect for interaction observed for total sleep time and average awaken time.

^c^ The pairwise comparison remained statistically significant after Holm correction.

^d^ The change in magnitude exceeded the clinical significance threshold defined by American Academy of Sleep Medicine (actigraphy tool: 5% in the sleep efficiency, 20 minutes in the wake time after sleep onset, and 2 times in the number of awakenings; subjective tool: 10% in the sleep efficiency, 30 minutes in the wake time after sleep onset, 30 minutes in the total sleep time).^18^

^e^ Mean values are averages across 7-day sleep diary. No effect for interaction observed for number of awakenings, sleep onset latency, and average awaken time.

### Secondary Outcomes

**Figure 2.**
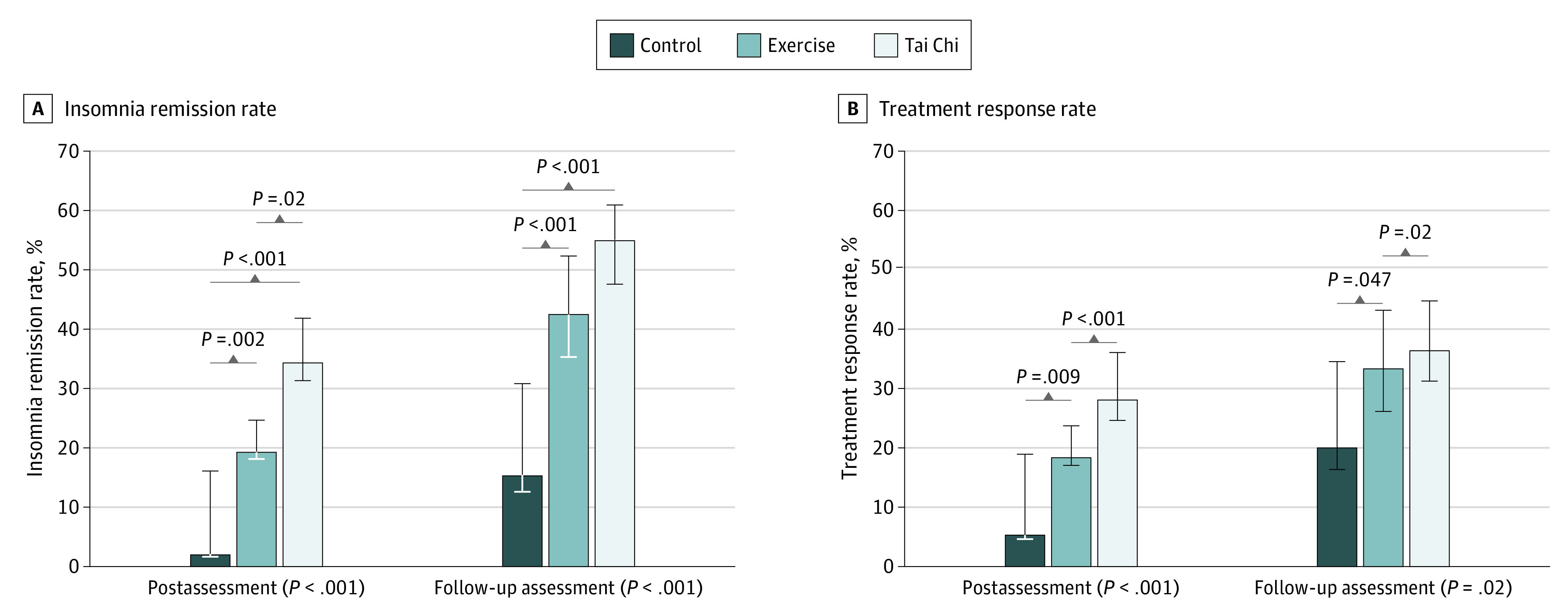
Insomnia Remission Rate and Treatment Response Rate (Change in Pittsburgh Sleep Quality Index Score by ≥5) Remission rates according to the *Diagnostic and Statistical Manual of Mental Disorders* (Fifth Edition) definition of chronic insomnia and treatment response rate are shown as a percentage of the observed cases. Error bars indicate range of remission/treatment response with the assumption that missing cases are all remitters/responders or nonremitters/nonresponders. Data were analyzed by logistic regression, followed by linear contracting for pairwise comparison analysis.

Compared with the control group, the insomnia remission rate was higher in the exercise and tai chi groups at postintervention (exercise vs control: 19.4% vs 2.1%; log odds, 2.4 [95% CI, 0.9-3.9]; *P* = .002; tai chi vs control: 34.4% vs 2.1%; log odds, 3.2 [95% CI, 1.7-4.6]; *P* < .001) and follow-up (exercise vs control: 42.5% vs 15.6%; log odds, 1.4 [95% CI, 0.7-2.1]; *P* < .001; tai chi vs control: 54.9% vs 15.6%; log odds, 1.9 [95% CI, 1.2-2.6]; *P* < .001). The tai chi group manifested a significantly larger insomnia remission rate than the exercise group at postintervention (34.4% vs 19.4%; log odds, 0.8 [95% CI, 0.1-1.4]; *P* = .02) but there was no significant difference between the exercise and tai chi groups at follow-up (Figure 2). Compared with the control group, the treatment response rate was higher in the exercise and tai chi groups at postintervention (exercise vs control: 18.4% vs 5.3%; log odds, 1.4 [95% CI, 0.4-2.4]; *P* = .009; tai chi vs control: 28.0% vs 5.3%; log odds, 1.9 [95% CI, 0.9-2.9]; *P* < .001) and follow-up (exercise vs control: 33.3% vs 20.0%; log odds, 0.7 [95% CI, 0.0-1.4]; *P* = .047; tai chi vs control: 36.3% vs 20.0%; log odds, 0.8 [95% CI, 0.2-1.5]; *P* = .02), with no significant differences between the exercise and tai chi groups at both assessments (Figure 2).

Interaction effects were observed in PSQI and ISI. Compared with the control group, both the exercise and tai chi groups demonstrated a decrease in PSQI at postintervention and follow-up, with a greater decrease in the tai chi group than the exercise group at postintervention but no significant difference between the groups at follow-up (Table 3). Compared with the control group, both the exercise and tai chi groups showed a reduction of ISI at postintervention and follow-up, with a greater reduction in the tai chi group than the exercise group at the postintervention but no significant difference was found between the exercise and tai chi groups at the follow-up (Table 3).

Interaction effects were observed in various parameters of the self-reported sleep diary, including sleep efficiency, wake time after sleep onset, and total sleep time. Compared with the control group, the exercise group showed no significant changes in sleep efficiency, wake time after sleep onset, and total sleep time at postintervention, but the tai chi group demonstrated favorable changes in subjectively measured sleep diary parameters (Table 3). Compared with the control group, both the exercise and tai chi groups showed an increase in sleep efficiency, wake time after sleep onset, and total sleep time at follow-up, with no significant difference between the 2 interventions (Table 3).

The subgroup analysis revealed an interaction effect in hypnotic medication usage. Compared with the control group, both the exercise and tai chi groups demonstrated a reduction in the use of hypnotic medication at both assessments but no significant differences were observed between the 2 interventions (Table 3).

## Discussion

The current study has incorporated both objective and subjective sleep assessments to (1) examine the effectiveness of tai chi for improving sleep by comparing with a passive control and (2) compare the effectiveness of tai chi with conventional exercise in older adults with insomnia. We found that both tai chi and conventional exercise led to improvements in actigraphy-assessed sleep parameters, including sleep efficiency, wake time after sleep onset, and number of awakenings, although the absolute improvements were modest. Both exercise modalities increased subjective sleep quality indicated by PSQI and ISI and reduced use of hypnotic medication compared with the control. Higher insomnia remission rate and treatment response rate were observed in both intervention groups. The beneficial effects on sleep from both intervention groups remained evident 24 months after experimental intervention, which can be ascribed to the self-motivated, regular practice of aerobic exercise and tai chi (eTable 2 in Supplement 2). The concurrent improvements in objective and subjective sleep and larger insomnia remission rate and treatment response rate support the potential of tai chi as an effective and feasible intervention for older adults with insomnia. Yet, the improvement in actigraphy-assessed objective sleep parameters are not significantly different between the 2 interventions, which does not support our hypothesis that tai chi induces more pronounced improvement in objective sleep than conventional exercise.

Existing studies with evidence that tai chi could ameliorate insomnia were based on subjective data.^7,19^ One merit of this study is the use of objective sleep assessment (ie, actigraphy) as the primary outcome. According to the clinical practice guidelines of the American Academy of Sleep Medicine, the clinical significance thresholds for sleep assessments measured by actigraphy are improvements by 5% in sleep efficiency, 20 minutes in the wake time after sleep onset, or 2 times in the number of awakenings.^18^ Accordingly, tai chi and conventional exercise both showed significant benefits in these clinical thresholds compared with the controls. There were improvements in the number of awakenings at both postintervention and follow-up assessments, and clinically significant changes in the wake time after sleep onset and sleep efficiency at the follow-up assessment. In the present study, the improvements in self-reported sleep parameters after tai chi intervention were not recapitulated by objective measures or vice versa (ie, total sleep time and number of awakening), which can be attributable to the inherent memory recall bias in self-reporting.^20^ While misperception of sleep is common in patients with insomnia, the perceived changes in overall well-being may partly account for the improved perception of sleep in response to tai chi intervention,^21^ and therefore further reiterate the importance of objective measurements by actigraphy in the present study to validate the therapeutic effects of tai chi. Previous attempts using polysomnography failed to identify improvements in objective sleep parameters following tai chi intervention.^5,19^ It is noteworthy that data collection by polysomnography is limited by its high operating cost, whereas more comprehensive insights can be made available by actigraphy through analyzing the data collected from 7 consecutive nights. Unlike actigraphy data collected directly from natural physiological rest in the current study, the combination of planned sleep schedules for polysomnography in previous studies and unfamiliar laboratory environments during polysomnography assessment may lead to sleep perturbation,^22^ and account for the discrepancies in the objectively assessed sleep outcomes between the previous studies and the current study.

The clinical practice guideline of the American College of Physicians recommends cognitive behavioral therapy for insomnia (CBT-I) as the first-line treatment for insomnia.^23^ Although CBT-I shows relatively fewer side effects than hypnotic medications, its high costs diminish greatly the availability in communities running on limited budgets. Because of the affordability and the absence of any known undesirable effects, physical exercise is a more feasible approach for managing insomnia from a public health perspective. Indeed, a 2012 meta-analysis showed that exercise is an effective approach to improve subjective sleep quality in middle-aged and older adults,^24^ and another study showed it to recapitulate the effects of hypnotic medications.^25^ Meta-analyses comparing effectiveness on improving subjective sleep outcomes reported moderate to large effect sizes for CBT-I (mean, 0.96; range, 0.46-1.44)^26^ and pharmacological treatment (mean, 0.87; range, 0.45-1.20).^26,27^ It is noteworthy that the effect sizes of tai chi in enhancing the subjective sleep quality (ie, 0.83 for PSQI; 0.92 for ISI) in the current work are larger than the average effect size of other behavioral interventions in older adults (ie, 0.76).^28^ The log odd ratio of the treatment response rate of tai chi relative to control was 1.9 as observed in this study, which can be translated as approximately 1.0 in term of effect size.^29^ Recent evidence also suggested that tai chi is not inferior to CBT-I in improving sleep in breast cancer survivors with insomnia.^5^ Considering that the improvements in various actigraphy-assessed sleep parameters have reached the threshold of clinically significant change, and the effect sizes for treatment response and the improvement in sleep quality after tai chi intervention are of large magnitudes and are comparable with approved insomnia managing approaches, the improvement in sleep after tai chi intervention is of clinical relevance and at the level of a minimally clinical important difference for insomnia severity. Taken together, the adoption of tai chi is an affordable alternative approach to manage insomnia in older adults who are unwilling or not able to participate in conventional exercise.

### Limitations

This study has several limitations. A limitation of this study is that the single-center setting may limit its generalizability. It is noteworthy that our participants were recruited from different districts of Hong Kong. We therefore believe that the characteristics of our participants are representative of the local population with insomnia, suggesting that our data can likely be translated to other geographical regions with similar demographic characteristics. Additionally, cultural differences and the acceptance of tai chi practice could limit its use. A 2020 study^10^ conducted worldwide have demonstrated the consistent beneficial effects of tai chi on subjective sleep, which suggests that tai chi could be feasibly implemented across cultural backgrounds. Hence, the results of this study are likely highly generalizable in many places around the globe.

## Conclusions

The tai chi intervention improved actigraphy-measured objective sleep parameters similar to conventional exercise, and the benefits remained after 24 months. The improvements in objective sleep parameters did not differ between the intervention groups. Although tai chi led to more evident improvements in subjective sleep parameters than conventional exercise upon completion of the intervention, the magnitudes of these benefits were not different between the intervention groups after 24 months. The concomitant improvements in objective and subjective sleep, as well as the larger insomnia remission and treatment response rates, support the notion that tai chi can be an alternative approach for insomnia management for older adults with insomnia.

## Notes

Supplement 1. **Study Protocol**

Supplement 2. **eTable 1.** Protocol of Interventions **eTable 2.** Self-Report of Time Spent on Different Exercise Modalities per Week at Follow-up Assessment

Supplement 3. **Data Sharing Statement**
