# Supplementary material for: Effects of Tai Chi or Exercise on Sleep in Older Adults With Insomnia: A Randomized Clinical Trial
Source: JAMA Netw Open. 2021 Feb 15;4(2):e2037199. doi: 10.1001/jamanetworkopen.2020.37199 (PMC7885034; doi:10.1001/jamanetworkopen.2020.37199)
Supplement: Supplement 1. — Study Protocol [file jamanetwopen-e2037199-s001.pdf]

Effects of Tai Chi or exercise on sleep in insomniac older adults: A three-arm randomized controlled trial

Study Protocol

Aim

This project aims to examine the efficacy of a 12-week Tai Chi intervention in improving sleep, especially in objective sleep assessed by actigraphy, in insomniac older adults by using a conventional exercise intervention as an active control.

Our Working Hypothesis

i) sleep outcome measures are significantly improved in insomniac elderly participants after receiving 12 weeks of Tai Chi training; ii) improvement of sleep outcome measures is found in Tai Chi group but not in passive control group; and iii) improvement of sleep outcome measures in Tai Chi group is greater than that in active control group, if any.

Background

Elderly insomnia is a serious public health problem. It has been estimated over 50% of elderly have sustained sleep complaints [1]. About 20-40% of the elderly worldwide are reported insomniac [2]. In Hong Kong, a high proportion (38%) of older people have been reported to have sleep disorders [3]. These figures are alarming because insomnia associates with co-morbidities including cognitive impairment, depression, mood/anxiety disorders, risks for falls, hypertension, and heart disease in elderly [4]. It also destructively affects daily functioning by impairing the memory and reducing the attention span and response time [2]. Most importantly, insomnia has been evidently shown to link with the increased risk of hospitalization and mortality [5]. As the proportion of geriatric population is rapidly increasing, it is foreseeable that the socioeconomic impact of elderly insomnia to the healthcare system will be undoubtedly aggravated.

Current conventional approaches for treating insomnia are not suitable or effective in elderly population. Pharmacologic treatment has always been a concern in insomniac senior patients due to the adverse drug effects including dependence, abuse, cognitive impairment and increase in risk of falls and hip fractures [6]. With fewer adverse effects and consistent efficacy, cognitive behavioral therapy for insomnia (CBTI) is taken as a more appropriate remedy for elderly insomnia. However, the operation of effective CBTI for insomnia is very labor demanding and cost-ineffective. The large-scale use of CBTI for elderly insomnia is not feasible due to the shortage of CBTI specialty-trained healthcare providers and the high treatment cost. The limited availability of CBTI treatment is far insufficient to match with the increasingly large demand of insomniac elderly. There is another problem with the conventional approaches. It is observed that most of the insomnia sufferers would not seek timely clinical consultation and they tend to initiate self-help treatments when facing the problem of insomnia [7]. This situation is suggested to be more common in Chinese elderly due to the traditional Confucius philosophy of reservation and quietness probably drive them more likely to keep the problem to themselves and become reluctant to seek prompt clinical help until serious medical symptom occurs. Given the prevalence of elderly insomnia is already high and keeps increasing, there is an urgent need to explore other effective therapeutic modalities preferably in forms of self-help remedies that can help to relieve the problem of elderly insomnia.

Tai Chi is a mind-body exercise that was simplified from a traditional form of Chinese martial art. It is a unique form of physical activity of low impact and slow body movement, which includes a meditation component. A number of beneficial effects of Tai Chi on geriatric health and fitness has been demonstrated. These health benefits include improvements of cardiorespiratory system, energy metabolism, immunity, muscular strength and balance [8]. In addition, Tai Chi exercise has been shown to improve psychological well-being via relieving symptoms of anxiety and depression and reducing mood disturbances [8]. Preliminary studies have provided evident that Tai Chi is beneficial in improving sleep in

older people. Favorable effects on sleep parameters have been reported in geriatric following 12 weeks to 6 months of Tai Chi training. The self-reported sleep quality is demonstrated to be improved by a 12-week of Tai Chi intervention in the senior residents in elderly home [9]. Favorable effects of 12-week of Tai Chi training in patients with chronic heart failure are reported by showing the enhancement of sleep stability as assessed by electrocardiogram-based sleep spectrogram [10]. A longer period (6-month) of Tai Chi training has also been shown to improve the Pittsburgh Sleep Quality Index (PSQI)-indicated sleep quality in community-dwelling older adults [11]. In older participants with moderate sleep complaint or sleep-disturbance, 24/25-week of Tai Chi training is shown to improve the self-rated sleep quality, habitual sleep efficiency, sleep duration, and sleep disturbance [12,13].

As a moderate type of exercise that is well perceived to be suitable for regular practice by older population, Tai Chi has advantages to be developed as self-help therapy of insomnia for older adults. It is expected that Tai Chi is more acceptable to the elderly patients to be incorporated with their daily life as an instant approach for remedying insomnia relative to the conventional clinical treatment such as CBTI. Additional advantages of Tai Chi include low-cost and can be conveniently practiced at any time and any place without requirement for extensive facilities. Certainly, the practice of Tai Chi is more accessible than the conventional CBTI treatment, which facilitates the large-scale use of Tai Chi to relieve insomnia in elderly population. Furthermore, Tai Chi can be practiced individually or in a group. If practicing in a group, Tai Chi provides additional benefit by serving as a vehicle for establishing social interaction through which older people can establish friendship and gain supports from other seniors.

All these preliminary data collectively indicate the beneficial effects of Tai Chi on improving sleep in geriatric population. Nonetheless, all these studies have a common design limitation, which is lack of the direct objective sleep measures. With the study limitation and research gaps, the present project is proposed to examine the therapeutic effects of Tai Chi on chronic insomnia in elderly by including objective sleep measures. The findings of this project are expected to have impact to unveil the efficacy of Tai Chi to alleviate elderly insomnia, which has been an epidemic healthcare problem that necessitates to be tackled promptly.

Sample Size Estimation

The effect size of Tai Chi intervention in older adults on sleep parameters assessed objectively by actigraphy or subjectively by 7-day sleep diary were not available by the time the present study was designed. Thus, the sample size estimation of this study is based on the published data on the effects of Tai Chi on subjective sleep outcome measures in older people. As this study employed a three-arm pretest-posttest design, estimation of sample size was originally performed using G*Power 3.0 by setting the test family section and statistical test section as “F tests” and “ANOVA: Repeated measures, within-between interaction”. An interaction effect size’ d 0.22 was calculated based on the subjective sleep quality of PSQI [12]. With 0.22 as the interaction effect size’ d (i.e. interaction effect size’ f = 0.11), 3 as number of groups, 2 as number of measurements, the calculation showed that 81 participants is needed to achieve a statistical power of 80% ($\alpha = 0.05$). The estimated sample size can be further justified by using a previously reported SMD in a systematic review study conducted by Sarris and Byrne, in which the modified cohen’s d of improvement in PSQI-assessed subjective sleep quality after Tai Chi intervention was 0.44. [14] The sample size estimation by using standardized mean difference as 0.44 in a two-tailed head-to-head test between two groups with alpha = 0.05 and power = 0.80, 83 participants per group is needed for a statistical power of 80% ($\alpha = 0.05$).

Participants

This study examines the efficacy of Tai Chi intervention on alleviating chronic insomnia in older adults by examining both objective and subjective sleep quality. Participants will be recruited via poster and leaflet distribution to community centers, elderly daycare centers, local universities, and large housing estates.

Inclusion Criteria of this study are: (1) be 60 years or older; (2) ethnic Chinese; (3) fulfill the Fifth Edition of *Diagnostic and Statistical Manual of Mental Disorders (DSM-5*) criteria for chronic insomnia including A)

manifestation of predominant, subpar sleep quantity or quality, B) exhibition of any sleep difficulties ranging from sleep initiation, sleep maintenance to early awakening with the inability in sleep restoration, C) concomitant presentation of significant distress or impairment, including fatigue or low energy, cognitive impairment, mood disturbance, behavioral problems, impaired occupational or academic functioning, impaired interpersonal or social functioning and negative impact on caregiver or family functioning, D) sleep difficulty occurs at least three nights per week, E) sleep difficulty has been persistent for at least three months, and F) sleep difficulty is not affected regardless of adequate opportunity given for sleep. Notably, this study intends to include the elderly suffered from all types of insomnia in order to enhance the generalizability and practical implication of our results.

<u>Exclusion Criteria</u> are set to exclude participants that are incapable to participate Tai Chi/conventional exercise and have major confounding conditions which are known to affect sleep. Exclusion criteria include: (1) cannot walk without assistive tool (e.g., cane); (2) any serious somatic condition that prevent participation for Tai Chi/conventional exercise activities; (3) current regular practicing of moderate exercise training or Tai Chi (>3 times a week of >30-min per session); (4) any serious chronic diseases known to affect sleep (e.g., cancer and autoimmune diseases); (5) under treatment of serious diseases known to affect sleep (e.g., cancer chemotherapy); (6) any chronic pain disorders known to affect sleep. Participants who are on sleep medications will not be excluded to increase the practical value of this study.

Study Design

<u>Participant Screening</u>

Participants who respond to our advertisements/promotion activities will undergo our screening process to confirm their eligibility for the study (i.e., older adults with chronic insomnia according to the DSM-5 criteria and do not fall into any exclusion criteria). Participants showing interest in participating in the study will be initially screened by brief self-reported questionnaires on their sleep and brief medical history via telephone. Potential participants will then be invited to attend a comprehensive in-person interview which includes diagnosis of chronic insomnia and comprehensive questionnaires to further obtain detailed sleep and medical history. Our research personnel will provide face-to-face explanation and assistance to the elderly participants in completing the questionnaires. Written informed consent will be obtained from all participants prior to the in-person interview. The interview will serve the purpose to obtain information to diagnose patients with chronic insomnia according to the DSM-5 criteria and to document the history of sleep-related abnormalities (e.g., apnea) and the use of sleep medications. Our clinical co-investigator plays a key consulting role in the interview supervision of the study. If there are any doubtful cases during the process of participant recruitment, decision is made based on the consultation with the clinical co-investigator.

<u>Randomization and Masking</u>

Eligible participants are randomly assigned to passive control (CON), active control (conventional exercise), or Tai Chi intervention. Randomization sequences are generated by using an automated permuted block algorithm with a block size of 30. Results of group allocation for the 30 participants will be respectively sealed in an envelope by an independent researcher. The envelopes will then be passed to the researcher who is responsible for participant enrollment. The envelopes that contained the group allocation results will not be opened until the participant enrollment process, thus concealing the randomized group allocation sequence from the researcher who is responsible for participant. During the recruitment process, the researcher who is responsible for participant enrollment will open an envelope in the chronological order and allocate each given eligible participant according to the randomization result enclosed. The same researcher will also be responsible to make appointments with the participants and the outcome assessors for taking the required measurements at each assessment time point. The allocated group of the participants will not be disclosed to the outcome assessors. Participants will be reminded not to disclose their group allocation to the outcome assessors such that the data collection process will be blinded.

<u>Outcome Measures</u>

In this study, the outcome measures will be conducted at baseline assessment (before intervention), post assessment (immediately after the completion of 12 weeks intervention), and follow up assessment (24 months after the end of intervention). Eligible participants will be invited to complete the baseline assessment and start the intervention no more than four weeks after the in-person interview.

Primary Outcome Measure

7-Day Actigraphy

Wrist actigraph (wGT3X-BT, Actigraph) will be used to objectively estimate sleep as movement and lack of movement correlate with wakefulness and sleep, respectively. This is a waterproof watch-like device that records physical movement by means of an accelerometer Participants will be instructed to wear our provided actigraph on the non-dominant wrist for 24-hour × 7 days. Actigraphy data will be extracted after the participants return the actigraph and a software provided by the manufacturer (ActilifeV6.11.7) will be used to analyze the data by sleep efficiency (total sleep time / total time in bed x 100%), wake time after sleep onset, number of awakenings per night, sleep onset latency, total sleep time, and average wake time per awakening.

Secondary Outcome Measures

7-Day Sleep Diary

Participants will be instructed to record their sleep pattern each morning for 7-day at all assessment time points in a provided sleep log that consists of questions to obtain information including bedtime, sleep rising time, wake time after sleep onset, total sleep time, number of awakenings, and sleep onset latency. Sleep efficiency will be estimated by (total sleep time / total time in bed) x 100%. Average awaken time will be estimated by (wake time after sleep onset / number of awakenings). Notably, the sleep diary has been shown to be a reliable instrument for collecting data about sleep/wake patterns, although it is a participative measure [15]. An acceptable percentage agreement between the sleep diaries data and polysomnographic data (kappa = 0.87) has been demonstrated previously [15].

Usage of Sleep Medication

The dosage and frequency of sleep aid medications such as narcotics, antihistamines (diphenhydramine), benzodiazepines (e.g., flurazepam, quazepam, triazolam, estazolam, temazepam, clonazepam, lorazepam, alprazolam, etc), and non-benzodiazepine, benzodiazepine receptor agonists (e.g., zolpidem, zaleplon, eszopiclone, etc) will be recorded in the 7-Day Sleep Diary.

Pittsburgh Sleep Quality Index (PSQI)

Pittsburgh Sleep Quality Index (PSQI) is a standardized instrument to estimate sleep quantity and quality. There are 19 items subjectively assessing sleep quantity and the perceived restfulness and disturbance of sleep by gathering information on usual bed time, wake time, time to fall asleep, time of actual sleep, and quality of sleep. Each item is rated on a “0-3” Likert scale, with higher score indicating poorer sleep quality. PSQI has been commonly used to discriminate people with normal sleep from those with primary insomnia. The Chinese version of PSQI will be used in this study. This Chinese version PSQI has been demonstrated to have satisfactory Cronbach’s alpha of 0.82-0.83 and test-retest reliability of 0.85 and has been adopted to study insomnia in Hong Kong Chinese older adults[16].

Insomnia Severity Index (ISI)

The Insomnia Severity Index (ISI) questionnaire will be used to measure the perceived insomnia severity. There are 7 items on the aspects of insomnia to be measured by this instrument and these include sleep-onset and sleep maintenance difficulties, satisfaction with the current sleep pattern, interference with daily functioning, noticeability of impairment due to sleep problems, degree of distress, and concern caused by sleep problems. Each item is rated on a “0-3” Likert scale, with higher score indicating more perceived severity of insomnia. The Chinese version ISI has been shown to have satisfactory content valid index of 0.94 and high internal consistency with Cronbach’s alpha of 0.81[17]. This Chinese version ISI has been demonstrated to be culturally relevant and psychometrically sound instrument for examining severity and impact of insomnia in Hong Kong Chinese older people [17].

Insomnia Remission Rate

Participants will undergo diagnosis of chronic insomnia at the post assessment and follow up assessment by a blinded assessor. Participants who are not meeting the DSM-5 criteria of chronic insomnia will be considered to reach remission from chronic insomnia.

Treatment Response

Treatment response is defined by a decrease in PSQI by at least 5 points, which indicates marked improvement or nearly complete or complete remission of insomnia symptoms. Treatment response will be measured at the post assessment and follow up assessment.

<u>Intervention</u>

Passive Control Group

Participants in CON received no intervention but monthly phone calls for recording the sleep conditions. These participants will be asked to continue their pre-existing usual care throughout the study period.

Active Control Group

Intervention of low-to-moderate intensity conventional exercise will be given to participants in active control group. Participants assigned to active control group are arranged with a conventional exercise training program conducted in small group (~13 participants with both males and females per class). Conventional exercise training is prescribed as a 12-week program, with three 1-hour instructor-led sessions per week. The conventional exercise classes are operated by certified fitness instructors. The 1-hour training session consists of 15-minute light static stretching exercises on major muscles, 15 to 20 minutes of body conditioning activities (e.g., arm curl, arm raise, should press, ¼ squat, heel raise and stepping on the ground), 15-minute walking or brisk walking exercise, and 10 to 15 minutes of cool down exercise (i.e. static stretching, deep breathing, and relaxing body movements). According to The Compendium of Physical Activities[18], the exercise intensity level of stretching exercise (PA code: 02101) is approximate to 2.3 metabolic equivalent (MET; 1 MET refers to the resting metabolic rate during quiet sitting, i.e., energy expenditure of 1 kcal or 4.184 kJ per kg body weight per hour), conditioning exercise with moderate effort (PA code: 02035) is approximate to 4.3 METs, walking with moderate pace (PA code: 17190) is approximate to 3.5, brisk walking (PA code: 17200) is approximate to 4.3 METs. To ensure the body exercise movements are practicable and safe particularly to elderly participants, all movements are kept simple and no additional weight is applied. Range of motion and numbers of repetition/set are adjusted for the ease of participants. The recruited fitness instructors will have to attend a briefing session to ensure that they understand the exact procedure of the conventional exercise intervention protocol. This training session is to make sure all fitness instructors are delivering the same fitness protocol throughout the study. Research personnel will occasionally visit the classes to monitor the fidelity of treatment protocol in the study.

The purpose of the active control group is to control for the effects of social gathering and moderate-intensity physical activity that would be parts of the Tai Chi treatment program. Interpretations are made based on the comparison between Tai Chi intervention and the two control groups (i.e., active and passive control groups). With the active control (conventional exercise) group, our study will be able to examine the specific effects of Tai Chi training itself by eliminating the factors such as group gathering, interactions among participants and with the class instructor, generic moderate-intensity physical activity, and the other factors resulting from operation of a small-group interventional program. Furthermore, this study design can also help to distinguish the specific effects of the “mind” component of Tai Chi (which is a mind-body exercise) by comparing the results obtained from Tai Chi group to the active control (conventional exercise) group in our project. Moreover, comparison made between Tai Chi and passive control groups will demonstrate the overall treatment effects of Tai Chi training when compared to “no intervention”. This comparison will demonstrate the effects of Tai Chi intervention over “no intervention”, which will be valuable in providing evidence for recommending Tai Chi as a therapeutic tool to physically sedentary elderly population.

Tai Chi Intervention Group

For the participants assigned to Tai Chi group, they will be arranged with a Tai Chi training program conducted in small group (~13 participants with both males and females per class). Tai Chi intervention is

prescribed as a 12-week program, with three 1-hour instructor-led sessions per week. The Tai Chi classes are operated by qualified/certified instructors. Each 1-hour training session consists of a brief warm-up stretching session followed by standard Tai Chi routine activities. The 24-form/movement simplified Yang style Tai Chi is adopted as it is the most popular form of Tai Chi [11]. The 24-form/movement simplified Yang style Tai Chi is adopted to reduce the complexity and time required and hence people could learn to practice within a relatively short period of time. This Yang style Tai Chi is suitable for most people including elderly to practice as it has been commonly adopted for older participants in the literature [11,19]. Similar to the conventional exercise, the Yang style Tai Chi is considered as a moderate-intensity exercise, with the metabolic expenditure approximate to 3.24 METs [20]. The recruited Tai Chi instructors will have to attend a briefing session to ensure that they understand the exact procedure of the Tai Chi intervention protocol. This training session is to make sure all Tai Chi instructors are delivering the same intervention protocol throughout the study. Research personnel will occasionally visit the Tai Chi classes to monitor the fidelity of treatment protocol in the study.

Venue for Tai Chi and Conventional Exercise Classes

Tai Chi and conventional exercise classes will be conducted in the open space areas of public recreational parks. In this study, public parks equipped with both open space and cover areas are selected. In case of inclement weather, classes will be moved to the cover areas of the same parks. Most of the classes will be conducted in the open space areas of the park except those on the days of inclement weather, which will be conducted in the cover area of the parks. The comparison made between Tai Chi and conventional exercise (active control) groups should be valid because the same set of venue arrangement for weather condition will be applied to both groups.

Plan to Emphasize Safety Issue

A compulsory safety briefing session will be arranged in the first class of Tai Chi and conventional exercise groups. In briefing session, safety precaution topic including safe training environment, safety regulation in the class, and response to emergency situation. Safety issues will be reinforced to instructors throughout the study.

Plan to Monitor Adverse Event

Adverse events will be monitored through reports from Tai Chi and fitness instructors, reports from participants. Instructors will be asked to fill a logbook after each of the Tai Chi/conventional exercise class to record any adverse events (such as falls or slips, fatigue, dizziness, headache, and knee strain injury, etc) indicated by participants that might be related to Tai Chi and conventional exercise training. Research personnel will contact the participants by telephone in Tai Chi group and conventional exercise group monthly to ask if they have any adverse events or safety issues that are related to their participation in the study. If sustained serious adverse events are found, clinical advice will be sought from our medical and nursing co-investigators.

Plan to Have Blind Outcome Assessor

The data collection for aforementioned outcome measures will be performed by the research personnel. It is noted that the front pages of all the questionnaires and sleep data record forms will display the names of participants for the research personnel and instructors to conduct the data collection. After the questionnaires and forms are collected from all groups at the beginning and the end of each of the sessions, all questionnaires and forms will go to the office of the Principal Investigator. The PI will then replace the participant name by a randomized code on the front pages of the questionnaires and forms. The participant identity of these codes will only be known to the PI but not the others during the process of data assessments which will be performed by other research personnel. The participant identity of the codes will finally be disclosed to the research personnel after the completion of the data assessments. This practice will ensure that the outcome assessors will be blinded to the group assignment of the data during assessments in order to avoid bias. Participants will be reminded not to disclose their received intervention to the assessors during the assessments.

Data Analysis

Data will be expressed as mean (standard deviation). Intention-to-treat analysis will be employed. Generalized estimated equation model with baseline measurement as covariate will be used to analyze the quantitative data. Pairwise comparison will be performed using closed test procedure with Holm-Bonferroni correction to detect statistical differences among groups. A sub-group analysis will be conducted in participants taking hypnotic medication to examine changes in hypnotic medication usage. Logistic regression will be performed to examine categorical data, followed by linear contracting for pairwise comparison analysis. All statistical analyses will be performed using R. Statistical significance will be accepted at $p < 0.05$.

References

1. Foley DJ, Monjan AA, Brown SL, Simonsick EM, Wallace RB, Blazer DG. Sleep complaints among elderly persons: an epidemiologic study of three communities. *Sleep.* 1995;18(6):425-432.
2. Schubert CR, Cruickshanks KJ, Dalton DS, Klein BE, Klein R, Nondahl DM. Prevalence of sleep problems and quality of life in an older population. *Sleep.* 2002;25(8):889-893.
3. Chiu HF, Leung T, Lam LC, et al. Sleep problems in Chinese elderly in Hong Kong. *Sleep.* 1999;22(6):717-726.
4. Stewart R, Besset A, Bebbington P, et al. Insomnia comorbidity and impact and hypnotic use by age group in a national survey population aged 16 to 74 years. *Sleep.* 2006;29(11):1391-1397.
5. Manabe K, Matsui T, Yamaya M, et al. Sleep patterns and mortality among elderly patients in a geriatric hospital. *Gerontology.* 2000;46(6):318-322.
6. Glass J, Lanctot KL, Herrmann N, Sproule BA, Busto UE. Sedative hypnotics in older people with insomnia: meta-analysis of risks and benefits. *BMJ.* 2005;331(7526):1169.
7. Morin CM, LeBlanc M, Daley M, Gregoire JP, Merette C. Epidemiology of insomnia: prevalence, self-help treatments, consultations, and determinants of help-seeking behaviors. *Sleep Med.* 2006;7(2):123-130.
8. Wang C, Collet JP, Lau J. The effect of Tai Chi on health outcomes in patients with chronic conditions: a systematic review. *Arch Intern Med.* 2004;164(5):493-501.
9. Hosseini H, Esfirizi MF, Marandi SM, Rezaei A. The effect of Ti Chi exercise on the sleep quality of the elderly residents in Isfahan, Sadeghieh elderly home. *Iranian journal of nursing and midwifery research.* 2011;16(1):55-60.
10. Yeh GY, Mietus JE, Peng CK, et al. Enhancement of sleep stability with Tai Chi exercise in chronic heart failure: preliminary findings using an ECG-based spectrogram method. *Sleep Med.* 2008;9(5):527-536.
11. Nguyen MH, Kruse A. A randomized controlled trial of Tai chi for balance, sleep quality and cognitive performance in elderly Vietnamese. *Clinical interventions in aging.* 2012;7:185-190.
12. Irwin MR, Olmstead R, Motivala SJ. Improving sleep quality in older adults with moderate sleep complaints: A randomized controlled trial of Tai Chi Chih. *Sleep.* 2008;31(7):1001-1008.
13. Li F, Fisher KJ, Harmer P, Irbe D, Tearse RG, Weimer C. Tai chi and self-rated quality of sleep and daytime sleepiness in older adults: a randomized controlled trial. *J Am Geriatr Soc.* 2004;52(6):892-900.
14. Sarris J, Byrne GJ. A systematic review of insomnia and complementary medicine. *Sleep Med Rev.* 2011;15(2):99-106.
15. Rogers AE, Caruso CC, Aldrich MS. Reliability of sleep diaries for assessment of sleep/wake patterns. *Nurs Res.* 1993;42(6):368-372.
16. Tsai PS, Wang SY, Wang MY, et al. Psychometric evaluation of the Chinese version of the Pittsburgh Sleep Quality Index (CPSQI) in primary insomnia and control subjects. *Qual Life Res.* 2005;14(8):1943-1952.
17. Yu DS. Insomnia Severity Index: psychometric properties with Chinese community-dwelling older people. *J Adv Nurs.* 2010;66(10):2350-2359.
18. Ainsworth BE, Haskell WL, Herrmann SD, et al. 2011 Compendium of Physical Activities: A Second Update of Codes and MET Values. *Medicine & Science in Sports & Exercise.* 2011;43(8):1575-1581.
19. Lee MS, Lee EN, Kim JI, Ernst E. Tai chi for Lowering Resting Blood Pressure in the Elderly: a Systematic Review. *J Eval Clin Pract.* 2010;16(4):818-824.

20. Hui SS, Woo J, Kwok T. Evaluation of energy expenditure and cardiovascular health effects from Tai Chi and walking exercise. *Hong Kong Med J.* 2009;15 Suppl 2:4-7.
