# Supplementary material for: Effects of Tai Chi or Exercise on Sleep in Older Adults With Insomnia: A Randomized Clinical Trial
Source: JAMA Netw Open. 2021 Feb 15;4(2):e2037199. doi: 10.1001/jamanetworkopen.2020.37199 (PMC7885034; doi:10.1001/jamanetworkopen.2020.37199)
Supplement: Supplement 2. — eTable 1. Protocol of Interventions eTable 2. Self-Report of Time Spent on Different Exercise Modalities per Week at Follow-up Assessment [file jamanetwopen-e2037199-s002.pdf]

Supplemental Online Content

Siu PM, Yu AP, Tam BT, et al. Effects of tai chi or exercise on sleep in older adults with insomnia: a randomized clinical trial. *JAMA Netw Open*. 2021;4(2):e2037199. doi:10.1001/jamanetworkopen.2020.37199

eTable 1. Protocol of Interventions
eTable 2. Self-Report of Time Spent on Different Exercise Modalities per Week at Follow-up Assessment

This supplemental material has been provided by the authors to give readers additional information about their work.

© 2021 Siu PM et al. *JAMA Network Open.*

eTable 1. Protocol of Interventions

Session	Tai Chi intervention (TC Group)	Conventional exercise intervention (EX Group)
1	• Introduction of Tai Chi, including the history, theory and basic working principle.<br>• 10 min breathing exercise<br>• 20 min relaxation exercise<br>• 20 min standing pose meditation	• Introduction of generic exercise without tools<br>• 10 min stretching<br>• 15 min stepping on the ground and arm curl and ¼ squat<br>• 15 min walking<br>• 15 min light static stretching, deep breathing activities and relaxing body movements
2	• 15 min breathing exercise<br>• 15 min relaxation exercise<br>• 15 min standing pose meditation<br>• 15 min basic technique of weight shifting and walking	• 15 min stretching<br>• 15 min stepping on the ground, arm curl and ¼ squat<br>• 15 min walking<br>• 15 min light static stretching, deep breathing activities and relaxing body movements
3	• 15 min warm-up exercise<br>• 20 min basic technique of weight shifting and walking<br>• 15 min learn "starting pose of the 24 forms"<br>• 10 min breathing and relaxation exercise	• 15 min stretching<br>• 15 min stepping on the ground, arm curl, shoulder press and ¼ squat<br>• 15 min walking<br>• 15 min light static stretching, deep breathing activities and relaxing body movements
4	• 15 min warm-up exercise<br>• 15 min learn and practice "starting pose of the 24 forms"<br>• 20 min learn "part the wild horse's mane"<br>• 10 min breathing and relaxation exercise	• 15 min stretching<br>• 15 min stepping on the ground, arm curl, shoulder press and ¼ squat<br>• 15 min brisk walking<br>• 15 min light static stretching, deep breathing activities and relaxing body movements
5	• 15 min warm-up exercise<br>• 25 min practice "part the wild horse's mane"<br>• 10 min learn "white crane spreads its wings"<br>• 10 min breathing and relaxation exercise	• 15 min stretching<br>• 15 min stepping on the ground, arm curl, shoulder press and ¼ squat<br>• 15 min brisk walking<br>• 15 min light static stretching, deep breathing activities and relaxing body movements
6	• 15 min warm-up exercise<br>• 35 min practice "part the wild horse's mane" and "white crane spreads its wings"<br>• 10 min breathing and relaxation exercise	• 15 min stretching<br>• 15 min stepping on the ground, arm curl, shoulder press and ¼ squat<br>• 15 min brisk walking<br>• 15 min light static stretching, deep breathing activities and relaxing body movements
7	• 15 min warm-up exercise<br>• 15 min practice previously learnt forms<br>• 20 min learn "brush knee and twist step"<br>• 10 min breathing and relaxation exercise	• 15 min stretching<br>• 15 min stepping on the ground, arm curl, punching and ¼ squat<br>• 15 min brisk walking<br>• 15 min light static stretching, deep breathing activities and relaxing body movements
8	• 15 min warm-up exercise<br>• 35 min practice previously learnt forms<br>• 10 min breathing and relaxation exercise	• 15 min stretching<br>• 15 min stepping on the ground, arm curl, punching and ¼ squat<br>• 15 min brisk walking<br>• 15 min light static stretching, deep breathing activities and relaxing body movements
9	• 15 min warm-up exercise<br>• 25 min practice previously learnt forms<br>• 10 min learn "playing the lute"<br>• 10 min breathing and relaxation exercise	• 15 min stretching<br>• 15 min stepping on the ground, arm curl, punching and ¼ squat<br>• 15 min brisk walking<br>• 15 min light static stretching, deep breathing activities and relaxing body movements
10	• 15 min warm-up exercise<br>• 15 min practice previously learnt forms<br>• 20 min learn "reverse reeling forearm"<br>• 10 min breathing and relaxation exercise	• 15 min stretching<br>• 15 min stepping on the ground, arm curl, punching and ¼ squat<br>• 15 min brisk walking<br>• 15 min light static stretching, deep breathing activities and relaxing body movements

© 2021 Siu PM et al. *JAMA Network Open.*

11	• 15 min warm-up exercise<br>• 35 min practice previously learnt forms<br>• 10 min breathing and relaxation exercise	• 15 min stretching<br>• 15 min stepping on the ground, arm curl, punching and ¼ squat<br>• 15 min brisk walking<br>• 15 min light static stretching, deep breathing activities and relaxing body movements
12	• 15 min warm-up exercise<br>• 15 min practice previously learnt forms<br>• 20 min learn "grasp sparrow's tail"<br>• 10 min breathing and relaxation exercise	• 15 min stretching<br>• 15 min stepping on the ground, arm curl, punching and ¼ squat<br>• 15 min brisk walking<br>• 15 min light static stretching, deep breathing activities and relaxing body movements
13	• 15 min warm-up exercise<br>• 35 min practice previously learnt forms<br>• 10 min breathing and relaxation exercise	• 15 min stretching<br>• 15 min stepping on the ground, arm swing, shoulder rotation, punching and ¼ squat<br>• 15 min brisk walking<br>• 15 min light static stretching, deep breathing activities and relaxing body movements
14	• 15 min warm-up exercise<br>• 25 min practice previously learnt forms<br>• 10 min learn "single whip"<br>• 10 min breathing and relaxation exercise	• 15 min stretching<br>• 15 min stepping on the ground, arm swing, shoulder rotation, punching and ¼ squat<br>• 15 min brisk walking<br>• 15 min light static stretching, deep breathing activities and relaxing body movements
15	• 15 min warm-up exercise<br>• 20 min practice previously learnt forms<br>• 15 min learn "wave hands like clouds"<br>• 10 min breathing and relaxation exercise	• 15 min stretching<br>• 15 min stepping on the ground, arm swing, shoulder rotation, punching and ¼ squat<br>• 15 min brisk walking<br>• 15 min light static stretching, deep breathing activities and relaxing body movements
16	• 15 min warm-up exercise<br>• 35 min practice previously learnt forms<br>• 10 min breathing and relaxation exercise	• 15 min stretching<br>• 15 min stepping on the ground, arm swing, shoulder rotation, punching and ¼ squat<br>• 15 min brisk walking<br>• 15 min light static stretching, deep breathing activities and relaxing body movements
17	• 15 min warm-up exercise<br>• 25 min practice previously learnt forms<br>• 10 min learn "high pat on horse"<br>• 10 min breathing and relaxation exercise	• 15 min stretching<br>• 15 min stepping on the ground, arm swing, shoulder rotation, punching and ¼ squat<br>• 15 min brisk walking<br>• 15 min light static stretching, deep breathing activities and relaxing body movements
18	• 15 min warm-up exercise<br>• 20 min practice previously learnt forms<br>• 15 min learn "right heel kick"<br>• 10 min breathing and relaxation exercise	• 15 min stretching<br>• 15 min stepping on the ground, arm swing, shoulder rotation, punching and ¼ squat<br>• 15 min brisk walking<br>• 15 min light static stretching, deep breathing activities and relaxing body movements
19	• 15 min warm-up exercise<br>• 35 min practice previously learnt forms<br>• 10 min breathing and relaxation exercise	• 15 min stretching<br>• 15 min stepping on the ground, arm swing, arm curl, arm abduction and ¼ squat<br>• 15 min brisk walking<br>• 15 min light static stretching, deep breathing activities and relaxing body movements
20	• 15 min warm-up exercise<br>• 25 min practice previously learnt forms<br>• 10 min learn "strike to ears with both fists"<br>• 10 min breathing and relaxation exercise	• 15 min stretching<br>• 15 min stepping on the ground, arm swing, arm curl, arm abduction and ¼ squat<br>• 15 min brisk walking<br>• 15 min light static stretching, deep breathing activities and relaxing body movements
21	• 15 min warm-up exercise<br>• 20 min practice previously learnt forms<br>• 15 min learn "turn body and left heel kick"<br>• 10 min breathing and relaxation exercise	• 15 min stretching<br>• 15 min stepping on the ground, arm swing, arm curl, arm abduction and ¼ squat<br>• 15 min brisk walking

© 2021 Siu PM et al. *JAMA Network Open.*

		• 15 min light static stretching, deep breathing activities and relaxing body movements
22	• 15 min warm-up exercise<br>• 35 min practice previously learnt form”<br>• 10 min breathing and relaxation exercise	• 15 min stretching<br>• 15 min stepping on the ground, arm swing, arm curl, arm abduction and ¼ squat<br>• 15 min brisk walking<br>• 15 min light static stretching, deep breathing activities and relaxing body movements
23	• 15 min warm-up exercise<br>• 15 min practice previously learnt forms<br>• 20 min learn “left lower body and stand on one leg” and “right lower body and stand on one leg”<br>• 10 min breathing and relaxation exercise	• 15 min stretching<br>• 15 min stepping on the ground, arm swing, arm curl, arm abduction and ¼ squat<br>• 15 min brisk walking<br>• 15 min light static stretching, deep breathing activities and relaxing body movements
24	• 15 min warm-up exercise<br>• 35 min practice previously learnt forms<br>• 10 min breathing and relaxation exercise	• 15 min stretching<br>• 15 min stepping on the ground, arm swing, arm curl, arm abduction and ¼ squat<br>• 15 min brisk walking<br>• 15 min light static stretching, deep breathing activities and relaxing body movements
25	• 15 min warm-up exercise<br>• 35 min learn “shuttle back and forth”<br>• 10 min breathing and relaxation exercise	• 15 min stretching<br>• 15 min stepping on the ground, arm swing, arm curl, punching and calf raise<br>• 15 min brisk walking<br>• 15 min light static stretching, deep breathing activities and relaxing body movements
26	• 15 min warm-up exercise<br>• 25 min practice previously learnt forms<br>• 10 min learn “needle at sea bottom”<br>• 10 min breathing and relaxation exercise	• 15 min stretching<br>• 15 min stepping on the ground, arm swing, arm curl, punching and calf raise<br>• 15 min brisk walking<br>• 15 min light static stretching, deep breathing activities and relaxing body movements
27	• 15 min warm-up exercise<br>• 20 practice previously learnt forms<br>• 15 learn “fan through back”<br>• 10 min breathing and relaxation exercise	• 15 min stretching<br>• 20 min stepping on the ground, arm swing, arm curl, punching and calf raise<br>• 15 min brisk walking<br>• 10 min light static stretching, deep breathing activities and relaxing body movements
28	• 15 min warm-up exercise<br>• 15 min practice previously learnt forms<br>• 20 min learn “turn body, deflect, parry, and punch”<br>• 10 min breathing and relaxation exercise	• 15 min stretching<br>• 20 min stepping on the ground, arm swing, arm curl, punching and calf raise<br>• 15 min brisk walking<br>• 10 min light static stretching, deep breathing activities and relaxing body movements
29	• 15 min warm-up exercise<br>• 35 min practice previously learnt forms<br>• 10 min breathing and relaxation exercise	• 15 min stretching<br>• 20 min stepping on the ground, arm swing, arm curl, punching and calf raise<br>• 15 min brisk walking<br>• 10 min light static stretching, deep breathing activities and relaxing body movements
30	• 15 min warm-up exercise<br>• 20 min practice previously learnt forms<br>• 15 min learn “appears closed”, “cross hands” and “closing”<br>• 10 min breathing and relaxation exercise	• 15 min stretching<br>• 20 min stepping on the ground, arm swing, arm abduction, shoulder press, calf raise and ¼ squat<br>• 15 min brisk walking<br>• 10 min light static stretching, deep breathing activities and relaxing body movements
31	• 15 min warm-up exercise<br>• 35 min practice previously learnt forms with refinement of posture, movement and meditation components<br>• 10 min breathing and relaxation exercise	• 15 min stretching<br>• 20 min stepping on the ground, arm swing, arm abduction, shoulder press, calf raise and ¼ squat<br>• 15 min brisk walking

© 2021 Siu PM et al. *JAMA Network Open.*

		• 10 min light static stretching, deep breathing activities and relaxing body movements
32	• 15 min warm-up exercise<br>• 35 min practice previously learnt forms with refinement of posture, movement and meditation components<br>• 10 min breathing and relaxation exercise	• 15 min stretching<br>• 20 min stepping on the ground, arm swing, arm abduction, shoulder press, calf raise and ¼ squat<br>• 15 min brisk walking<br>• 10 min light static stretching, deep breathing activities and relaxing body movements
33	• 15 min warm-up exercise<br>• 35 min practice previously learnt forms with refinement of posture, movement and meditation components<br>• 10 min breathing and relaxation exercise	• 15 min stretching<br>• 20 min stepping on the ground, arm swing, arm abduction, shoulder press, calf raise and ¼ squat<br>• 15 min brisk walking<br>• 10 min light static stretching, deep breathing activities and relaxing body movements
34	• 15 min warm-up exercise<br>• 35 min practice previously learnt forms with refinement of posture, movement and meditation components<br>• 10 min breathing and relaxation exercise	• 15 min stretching<br>• 20 min stepping on the ground, arm swing, arm abduction, shoulder press, calf raise and ¼ squat<br>• 15 min brisk walking<br>• 10 min light static stretching, deep breathing activities and relaxing body movements
35	• 15 min warm-up exercise<br>• 35 min practice previously learnt forms with refinement of posture, movement and meditation components<br>• 10 min breathing and relaxation exercise	• 15 min stretching<br>• 20 min stepping on the ground, arm swing, arm abduction, shoulder press, calf raise and ¼ squat<br>• 15 min brisk walking<br>• 10 min light static stretching, deep breathing activities and relaxing body movements
36	• 15 min warm-up exercise<br>• 35 min practice previously learnt forms with refinement of posture, movement and meditation components<br>• 10 min breathing and relaxation exercise	• 15 min stretching<br>• 20 min stepping on the ground, arm swing, arm abduction, shoulder press, calf raise and ¼ squat<br>• 15 min brisk walking<br>• 10 min light static stretching, deep breathing activities and relaxing body movements

The warm-up exercise of Tai Chi intervention includes breathing exercise, relaxation exercise, standing pose meditation. The training sessions were operated by certified fitness instructors/Tai Chi masters, who had at least 5 years of teaching experience in older adults. The performance of the participants was continuously and closely monitored by the instructors. Adjustments of intensity, range of motion and numbers of repetitive exercises were made when deemed necessary by the instructors to assure practicing safe exercise program.

© 2021 Siu PM et al. *JAMA Network Open.*

eTable 2. Self-Report of Time Spent on Different Exercise Modalities per Week at Follow-up Assessment

	CON	EX	TC
Stretching (min)	34.2 (68.8)	54.3 (86.0)	30.8 (54.9)
Aerobic Exercise (min)	61.5 (138.9)	252.9 (152.6)	61.6 (120.9)
Muscle Strengthening Activity (min)	6.3 (36.9)	12.1 (39.2)	6.9 (70.3)
Mind-body Exercise (min)	4.7 (26.9)	55.2 (107.1)	224.3 (145.1)

All values are expressed as mean (standard deviation). CON; control group, EX; conventional exercise group, TC; Tai Chi group. All participants were required to report the time spent performing any physical exercise in the 7-day period prior to the follow-up assessment. Participants in EX and TC spent more than 300 minutes exercising per week, whereas participants in CON exercised for approximately 100 minutes per week.

© 2021 Siu PM et al. *JAMA Network Open.*
