# Supplementary material for: Effects of Tai Chi or Exercise on Sleep in Older Adults With Insomnia: A Randomized Clinical Trial
Source: JAMA Netw Open. 2021 Feb 15;4(2):e2037199. doi: 10.1001/jamanetworkopen.2020.37199 (PMC7885034; doi:10.1001/jamanetworkopen.2020.37199)
Supplement: Supplement 3. — Data Sharing Statement [file jamanetwopen-e2037199-s003.pdf]

Siu PM, Yu AP, Tam BT, et al. Effects of tai chi or exercise on sleep in older adults with insomnia: a randomized clinical trial. *JAMA Netw Open*. 2021;4(2):e2037199. doi:10.1001/jamanetworkopen.2020.37199

Data Sharing Statement

Data

Data available: Yes
Data types: Deidentified participant data
How to access data: Individual participant data used in the reported results have been de-identified (text, tables, and figures). The data, study protocol, and the statistical analysis plan will be shared beginning 3 months and ending 5 years following the publication of this Article. Data will be shared with researchers who provide a methodologically sound proposal with achievable aims. Proposals should be directed to pmsiu@hku.hk, and those requesting access to the data will need to sign a data-access agreement.
When available: With publication

Supporting Documents

Document types: None

Additional Information

Who can access the data: Data will be shared with researchers who provide a methodologically sound proposal with achievable aims.
Types of analyses: Data will be shared with researchers who provide a methodologically sound proposal with achievable aims.
Mechanisms of data availability: Proposals should be directed to pmsiu@hku.hk, and those requesting access to the data will need to sign a data-access agreement.
